# The Bioavailability, Extraction, Biosynthesis and Distribution of Natural Dihydrochalcone: Phloridzin

**DOI:** 10.3390/ijms22020962

**Published:** 2021-01-19

**Authors:** Lei Tian, Jianxin Cao, Tianrui Zhao, Yaping Liu, Afsar Khan, Guiguang Cheng

**Affiliations:** 1 Faculty of Agriculture and Food, Kunming University of Science and Technology, Kunming 650500, China; leotian@kust.edu.cn (L.T.); jxcao321@hotmail.com (J.C.); food363@163.com (T.Z.); yapingliu820203@163.com (Y.L.); 2 Department of Chemistry, Abbottabad Campus, COMSATS University Islamabad, Abbottabad 22060, Pakistan; afsarhej@gmail.com

**Keywords:** phloridzin, pharmacological activities, physiological effects, medicine, food

## Abstract

Phloridzin is an important phytochemical which was first isolated from the bark of apple trees. It is a member of the dihydrochalcones and mainly distributed in the plants of the *Malus* genus, therefore, the extraction method of phloridzin was similar to those of other phenolic substances. High-speed countercurrent chromatography (HSCCC), resin adsorption technology and preparative high-performance liquid chromatography (HPLC) were used to separate and purify phloridzin. Many studies showed that phloridzin had multiple pharmacological effects, such as antidiabetic, anti-inflammatory, antihyperglycaemic, anticancer and antibacterial activities. Besides, the physiological activities of phloridzin are cardioprotective, neuroprotective, hepatoprotective, immunomodulatory, antiobesity, antioxidant and so on. The present review summarizes the biosynthesis, distribution, extraction and bioavailability of the natural compound phloridzin and discusses its applications in food and medicine.

## 1. Introduction

Natural products, especially from medicinal plants or herbs, have played a key role in human health and welfare. According to their chemical structures, they are usually classified as polyphenols (e.g., flavonoids, lignans, stilbenes, phenolic acids), alkaloids, sterols, terpenoids, polysaccharides and so on. These diverse phytochemical groups exhibit an extensive range of human health benefits, and are important sources of useful bioactive compounds. In fact, some of them have been taken as lead compounds for structural modifications or developed as modern clinical drugs. In addition, plant-based foods provide us with essential nutrients and contribute to a remarkable portion of our daily diets, including fruits, vegetables, beans, grains and nuts. Extensive studies have demonstrated that these diets are rich in dietary phytochemicals, which are recognized for their health benefits or medicinal properties as nutraceuticals. Recently, more and more people wish to consume the foods containing nutraceuticals for the improvement of the quality of human life and with the desired health potentials rather than to take medicine for illness prevention.

In the last two decades, many researchers focused on the isolation and identification of phytochemicals with novel structures and unique biological activities, but only 15% of them have been analyzed phytochemically, and only 6% have been used in biological screens [1]. To date, natural compounds have been widely used for keeping good health and treating various diseases, as agents of dietary supplements and pharmaceuticals. Lots of experimental results have demonstrated that natural products have various promising activities, such as antioxidant, anti-inflammatory, antimicrobial, antidiabetic, antiseptic, spasmolytic, sedative, hepatoprotective and immunomodulatory activities [2,3,4]. Moreover, numerous phytochemicals with a large range of biological and pharmacological activities have been extracted and identified from natural plants.

**Figure 1:**
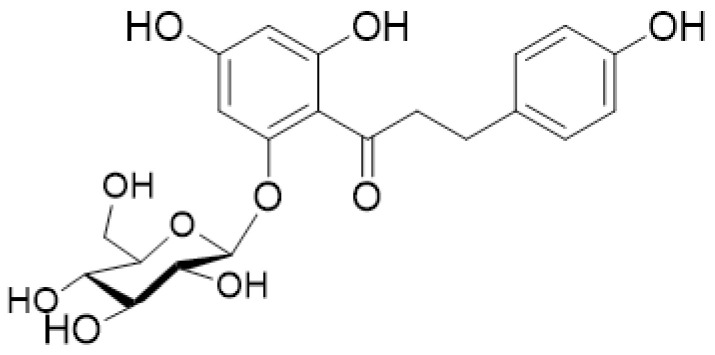
The structure of phloridzin.

Apple is one of the most commonly consumed fruits all over the world, and is a good source of polyphenols. There is a lot of evidence that frequent apple consumption contributes to human health, because polyphenols in apple have lots of beneficial biological functions, and phloridzin is one of polyphenols [5]. Phloridzin, also known as phlorizoside or phlorrhizin, belongs to the class of organic compounds which are known as flavonoid *O*-glycosides. Flavonoid O-glycosides are compounds containing a carbohydrate moiety, which is *O*-glycosidically linked to the 2-phenylchromen-4-one flavonoid backbone. Phloridzin (also referred to as phlorizin; chemical name phloretin-2′-*O*-β-d-glucopyranoside) is a glucoside of phloretin, a member of dihydrochalcone, a family of bicyclic flavonoids, and a monosaccharide derivative [6], which in turn is a subgroup in the diverse phenylpropanoid synthesis pathway in plants. Phloridzin is an extremely weak basic (essentially neutral) and bitter-tasting compound, with a molecular weight of 436.4 g/mol and the molecular formula C_21_H_24_O_10_ (Figure 1: 1-Propanone, 1-[2-(β-d-glucopyranosyloxy)-4,6-dihydroxyphenyl]-3-(4-hydroxyphenyl)-).

In 1835, a French scientist first isolated phloridzin from the bark of apple trees [7]. Phloridzin was previously considered a candidate for the treatment of fever, infectious diseases and malaria at that time [8]. Decades later, it was discovered that phloridzin could inhibit renal glucose reabsorption and causes glucosuria [9]. Some studies revealed that phloridzin was a competitive inhibitor of sodium–glucose cotransporters SGLT1 and SGLT2, because it could bind with the carrier that competed with d-glucose and, consequently, this reduced renal glucose transport, increased urinary glucose excretion and ameliorated plasma glucose concentration [10,11,12]. For decades, an enormous number of studies have been carried out on the pharmacological activities and physiological effects of phloridzin and its derivatives. This review aims to exhaustively sum up phloridzin’s biosynthesis, distribution, extraction and physiological and pharmacological effects.

## 2. Biosynthesis and Distribution of Phloridzin

The biosynthesis of phloridzin is different from general flavonoids, and its precursors are malonyl-CoA and p-coumaroyl-CoA. First, p-coumaroyl-CoA generates 4-hydroxydihydrocinnamoyl-CoA via the NADPH pathway, then malonyl-CoA and 4-hydroxydihydrocinnamoyl-CoA produce phloretin under the action of chalcone synthase, and the attachment of a glucose moiety to phloretin at position 2′ is the final step in the formation of phloridzin [13]. Two glycosyltransferases (both MdUGT88F1 and its paralog MdUGT88F4) convert phloretin to phloridzin in *Malus* plants [14,15]. Experimental evidence suggests that enoyl reductase-like genes-3 (ENRL-3) and ENRL-5 might contribute to the biosynthesis of phloridzin in apple [16].

Phloridzin is a member of the chalcone class of organic compounds and is mainly distributed in the plants of the *Malus* genus, although other plant species are described as containing phloridzin (Table 1). In humans, phloridzin is involved in lactose degradation. In addition, phloridzin is usually detected in higher concentrations in some foods, such as Mexican oregano, European plums and apples, while in pomegranates and apricots, it is found in lower concentrations. Phloridzin has also been found in several other foods such as epazotes, durians, Chinese broccoli, sesame and sweet potatoes. This could make phloridzin a potential biomarker for the consumption of these foods.

## 3. Extraction, Separation and Purification of Phloridzin

Phloridzin is solid at room temperature and poorly soluble in ether and cold water but soluble in ethanol and hot water, the melting point of phloridzin is 110 °C and it decomposes above 200 °C. Phloridzin’s extraction method is similar to that of other phenolic substances. Alberti et al. [47] reported that response surface methodology is an adequate approach for phloridzin extraction from apples, and phloridzin had higher yields (48.4%) in extractions with methanol. Zhang et al. [48] found that an ultrasound-assisted aqueous two-phase extraction strategy had significant advantages, including lower ethanol consumption, lower impurity of sugar and protein and higher extraction efficiency of phloridzin than the traditional solvent extraction with 35% and 80% ethanol. Paudel et al. [42] reported that phloridzin of black raspberry (*Rubus occidentalis* L.) fruits was extracted in ethyl acetate, isolated by semipreparative analytical HPLC and analyzed by nuclear magnetic resonance (NMR), HPLC–electrospray ionization mass spectrometry (ESI-MS) and ESI-MS/MS techniques.

At present, chemical extraction, high-speed countercurrent chromatography (HSCCC), resin adsorption technology and preparative HPLC methods are mainly used to separate and purify phloridzin. Dong et al. [32] separated, analyzed and identified 99.87% pure phloridzin from the crude extract of *Lithocarpus polystachyus* by thin layer chromatography (TLC) and HPLC-ESI-MS. Fromm et al. [49] reported that resin adsorption technology was a very effective tool for further purification as well as for the selective enrichment of phloridzin from apple seeds. Sun et al. [50,51] found that X-5 resin and polyamide resin were feasible and effective methods for the separation and purification of phloridzin from thinned young apples. Liang et al. [52] found that the HSCCC–HPLC–diode array detector–mass spectrometry (HSCCC-HPLC-DAD-MS) method served as a simple, rapid and effective way to achieve phloridzin with high purity (over 99%) from *Malus doumeri* leaves. Gao et al. [53] demonstrated that the prepared magnetic molecularly imprinted polymers (MMIPs) were suitable for phloridzin’s selective adsorption from complex samples such as natural medical plant extracts and biological samples. Li et al. [54] reported that macroporous resin followed by a preparative HPLC method was used for the purification of phloridzin from apple leaves; the phloridzin purity reached above 98% after further recrystallization with a recovery yield of 75.8%.

## 4. Therapeutic Properties of Phloridzin

Extensive research on phloridzin has been conducted for all kinds of purposes in medicine and biology since it was discovered in the bark of apple trees.

### 4.1. Antihyperglycemic Effect

Phloridzin improved hyperglycemia without altering insulin secretion in diabetic rats [55,56], because it could inhibit intestinal glucose uptake via sodium-dependent glucose transporters (SGLTs) and similarly control renal glucose reabsorption [57,58,59]. Malatiali et al. [60] reported that phloridzin induced normalization of blood glucose and prevented proteinuria, hyperfiltration and kidney hypertrophy, but not glomerular hypertrophy, in diabetic rats. Masumoto et al. [61] found that dietary phloridzin decreased the overexpression of SGLT1, cytochrome P450 2b10 (Cyp2b10) and epoxide hydrolase 1 (Ephx1) in the small intestine of diabetic mice and, accordingly, improved abnormal elevations in blood glucose levels. Osorio et al. [62] reported that phloridzin treatment reduced hyperglycemia, normalized hypertension and inhibited SGLT2 activity but did not modify SGLT2 expression in brush border membrane vesicles. Kobori et al. [63] revealed that dietary phloridzin significantly suppressed blood glucose levels, however, high continuous intakes of phloridzin reduced hepatic gene expressions related to metabolism, such as the citrate cycle, gluconeogenesis, fatty acid metabolism and branched chain amino acid metabolism in healthy mice. Najafian et al. [64] reported that phloridzin not only reduced blood glucose levels, but also improved lipid metabolism in streptozotocin-induced diabetic rats. Makarova et al. [65] demonstrated that unripe apple preparation containing phloridzin reduced postprandial glycemia and improved the health of diabetic patients. Mei et al. [66] found that phloridzin normalized the hyperglycemia of type 2 diabetes (db/db) mice by a decrease in serum lipopolysaccharides and gut microbiota changes. Wang et al. [67] reported that phloridzin accelerated liver glycogen synthesis, decreased hepatic gluconeogenesis and had hypoglycemic effects in type 2 diabetes mellitus mice. Lv et al. [68] reported that phloridzin from tea crabapple (*Malus hupehensis*) had a significant concentration-dependent inhibitory effect on α-glucosidase in vitro.

Despite the antihyperglycemic effects of phloridzin, it has some limitations, including poor absorption, rapid degradation and low bioavailability [7]. Phloridzin itself is not developed as a drug but, currently, some phloridzin-derived SGLT2 inhibitors (dapagliflozin, canagliflozin, empagliflozin, ertugliflozin, tofogliflozin and luseogliflozin) have gained approval in some developed countries [10].

### 4.2. Antioxidant Activity

Phloridzin acts as natural antioxidant, scavenges free radicals, inhibits lipid peroxidation, increases the activities of antioxidant enzymes and prevents oxidative stress (Table 2). Ma et al. [34] reported that the extracts from lotus seed epicarp (containing phloridzin) have antioxidant activity, which was identified by 2,2-diphenyl-1-picrylhydrazyl (DPPH) and 2,2-azino-bis(3-ethylbenzothiazoline-6-sulphonic acid) (ABTS) radical-scavenging methods. Similarly, Xiao et al. [69] demonstrated that phloridzin of *Malus domestica* exhibited antioxidant capacity by DPPH and ABST assays. Liaudanskas et al. [70] reported that apple leaf ethanol extract (phloridzin was the major compound) possessed strong antioxidant activity by ABTS, DPPH and ferric-reducing antioxidant power (FRAP) assays.

In addition to free radical-scavenging properties, phloridzin has been identified as a potent antioxidant in the inhibition of lipid peroxidation. Rupasinghe and Yasmin [71] reported that phloridzin had a significant effect in preventing peroxyl radical-induced oxidation by polyunsaturated fatty acid in aqueous emulsions. Thilakarathna et al. [72] found that phloridzin extracted from apple peel effectively inhibited human low density lipoprotein (LDL) cholesterol oxidation in vitro. Wang et al. [73] demonstrated that apple phloridzin extended the life span, improved the viability, alleviated the mortality rate induced by paraquat and H_2_O_2_ and increased the activity of antioxidant enzymes in fruit flies. Fan et al. [74] found that phloridzin was the main component of E Se tea extracts, which significantly prevented oxidative stress damage and reduced the apoptosis of H_2_O_2_-induced HepG2 cells.

The antioxidant activity of phloridzin was associated with other biological properties. Choi et al. [24] revealed that phloridzin had antioxidant activity, which affected stem cell fate in the skin via the inhibition of miR135b and following the synthesis of type IV collagen of the basement membrane. Sun et al. [75] reported that young apple polyphenols, mainly composed of phloridzin, showed strong antioxidant activity in vitro and a preservative effect on grass carp surimi during cold storage.

### 4.3. Anti-Inflammatory Effects

The anti-inflammatory activity of phloridzin has been investigated in some laboratory-based studies (Table 3). Shin et al. [76] found that phloridzin suppressed plasma pro-inflammatory adipokine levels and, furthermore, attenuated inflammation in diet-induced obese mice. Zhao et al. [77] reported that phloridzin metabolites decreased nitric oxide (NO) production and the expression of inducible nitric oxide synthase (iNOS) in lipopolysaccharide (LPS)-stimulated RAW264.7 cells, which showed anti-inflammatory activity. Zielinska et al. [78] demonstrated that apple phloridzin improved the quality of anti-inflammatory response in the intestine and, accordingly, ameliorated cytokine-driven inflammation. Chang et al. [79] reported that the metabolite of phloridzin suppressed the inflammatory response in RAW264.7 murine macrophages that had been stimulated by LPS from Gram-negative bacteria. Zhai et al. [80] showed that phloridzin decreased the expression of ultraviolet B (UVB)-induced pro-inflammatory cytokines and reduced acute inflammation infiltration in UVB-exposed skin. Tian et al. [81] showed that phloridzin significantly reduced the concentration of serum and adipose tissue pro-inflammatory cytokines and attenuated adipose tissue inflammation in high-fat diet (HFD)-fed mice.

### 4.4. Hepatoprotective Effects

Many reports suggested that phloridzin has a hepatoprotective function by regulating lipid metabolism and oxidative stress and inhibiting hepatic inflammation and apoptosis. Lu et al. [82] found that phloridzin decreased body weight gain and the levels of glucose, blood total cholesterol (TC) and blood triglycerides (TG) in blood and, accordingly, ameliorated the hepatic damage of type 2 diabetic mice. Shin et al. [76] reported that phloridzin also alleviated hepatic steatosis, inflammation and fibrosis by decreasing white adipose tissue and collagen accumulation. Khalifa et al. [83] elucidated the protective effects of phloridzin against methotrexate-induced hepatic injury in rats by the mitigation of oxidative stress, inflammation and apoptosis in hepatic tissues. Parathodi Illam et al. [84] reported that a combination of aqueous extracts of fruits (containing phloridzin) improved hepatic and renal glutathione peroxidase (GPx), superoxide dismutase (SOD) and catalase activities and glutathione (GSH) levels in normal Swiss albino mice. Wang et al. [85] demonstrated that apple phloridzin increased cell viability, relieved deoxyribonucleic acid (DNA) damage, oxidative stress and apoptosis in H_2_O_2_-induced HepG2 cells by the nuclear factor E2-related factor 2 (Nrf2) signaling pathway and anti-apoptosis genes. David-Silva et al. [86] found that phloridzin restored glycemic control and hepatic glucose metabolism, inhibited hepatic glucose production and ameliorated non-alcoholic fatty liver disease in type 2 diabetic mice.

### 4.5. Antitumor Effects

Phloridzin was utilized to trigger cancer cell death through either direct or indirect pathways. Qin et al. [87] showed that phloridzin from crabapple leaves had antitumor effects and, moreover, its derivatives had a stronger protective effect against the four tested tumor cell lines, which suggested that phloridzin and its derivatives could be considered as possible therapeutic agents against cancer. Aguiniga-Sanchez et al. [44] reported that a fruit methanol extract of *Sechium edule* var. *nigrum spinosum* contained phloridzin, and the extract eliminated tumor cells while protecting normal bone marrow cells.

### 4.6. Antibacterial Activity

In some experimental models, phloridzin exhibited antibacterial and antifungal activity. Sowa et al. [88] observed that extracts of *Malus domestica* leaves (containing a large amount of phloridzin) had antibacterial and antifungal activity by the inhibition of *Staphylococcus aureus* and *Enterococcus faecalis* and the fungus *Candida glabrata*. Lopes et al. [89] found that phloridzin was effective in inhibiting biofilm formation in *Staphylococcus aureus* strains, which increased efflux protein genes. Oleszek et al. [90] reported that extracts of apple pomace (fraction containing phloridzin) had antifungal activity by the inhibition of mycotoxigenic fungal growth.

### 4.7. Cardioprotective Effects

Phloridzin was efficient in protecting the heart in certain diseases. Lee et al. [26] reported that extracts of *Glycine max* seed coat contained a large amount of phloridzin, and the extracts attenuated the adhesion of THP-1 to LPS-stimulated human umbilical vascular endothelial cells, which suggested that phloridzin was a potential coronary heart disease preventive agent. Cai et al. [91] identified differentially expressed proteins involved in cardiac lipid metabolism, mitochondrial function and cardiomyopathy by isobaric tags for relative and absolute quantitation (iTRAQ) proteomics, which implied that phloridzin may prevent the development of diabetic cardiomyopathy by regulating the expression of key proteins in these processes. Hirose et al. [92] indicated that phloridzin prevented ischemia-induced ventricular tachyarrhythmia through the improvement of impulse conduction slowing during ischemia in Langendorff-perfused guinea pig hearts.

### 4.8. The Effects of Phloridzin on Bone

In addition to those activities discussed above, phloridzin also exhibited a capacity to improve bone in vivo. Puel et al. [93] reported that phloridzin was able to elicit protective effects on bone loss in osteoporosis in relation to inflammation. Antika et al. [94] reported that phloridzin and phloretin could promote osteoblastogenic bone formation through activating canonical glycogen synthase kinase3β (GSK-3β)/β-catenin signaling involving runt-related transcription factor 2 (Runx2) in cell-based and aged mouse models. Londzin et al. [95] found that phloridzin (20 mg/kg) significantly increased unfavorable effects on the muscle and decreased the growth of bones, whereas it (50 mg/kg) did not affect most of the detected musculoskeletal parameters in type 2 diabetic rats.

### 4.9. Other Effects

Phloridzin had been reported to have a variety of other biological activities. Andlauer et al. [96] found that the natural substance phloridzin (present in apples) improved genistin absorption in isolated rat small intestine. Jung et al. [97] demonstrated that phloridzin acted through the cAMP pathway to increase tyrosinase transcriptional activities, thereby leading to the stimulation of melanogenesis. Gatidis et al. [98] revealed that a novel effect of phloridzin was the blunting of eryptosis, following energy depletion and oxidative stress. Xiang et al. [99] presented evidence that phloridzin had antiaging effects on yeast by increasing the activity of superoxide dismutase (SOD) and SIRT1. Wang et al. [100] reported that phloridzin inhibited polycystic kidney disease progression by the MAPK signaling pathway in a rat model. Pei et al. [101] revealed that phloridzin may be a renal protective agent by regulating differentially expressed proteins, which not only affected glomerular dysfunction and tubular transport, but also played active roles in oxidative stress and lipid metabolism. Wang et al. [102] demonstrated that phloridzin increased plasma lipoprotein lipase activity, and improved triglyceride metabolism via the adenosine 5‘-monophosphate (AMP)-activated protein kinase (AMPK) pathway in stress-loaded mice. Wang et al. [103] found that extracts from the fruits of *Malus baccata* contained phloridzin, and the extracts had radioprotective and immunomodulatory activities. Kanda et al. [104] found that phloridzin strongly decreased high K^+^-induced contraction in phasic muscle (tenia coli), but slightly affected tonic muscle (trachea) because of the inhibition of energy metabolism via SGLT1. Li et al. [105] found that phloridzin increased NO output, suppressed SGLT1 and SGLT2 expression, and promoted the consumption of glucose, which ameliorated endothelial dysfunction by the phosphatidylinositol 3-kinase (PI3K)/protein kinase B (AKT)/endothelial nitric oxide synthase (eNOS) signaling pathway in palmitic acid-induced human umbilical vein endothelial cells. Kumar et al. [106] reported that phloridzin abrogated the Cdk5-mediated phosphorylation of peroxisome proliferator activated-receptor γ (PPARγ) at the ser273 site by strongly inhibiting Cdk5 activation, which increased insulin sensitivity and glucose uptake in differentiated adipocytes. Park et al. [107] found that phloridzin treatment resulted in the enhancement of erectile function, which was similar to that seen with insulin therapy, but phloridzin did not show anabolic effects, such as weight gain, in diabetic rats.

**Figure 2:**
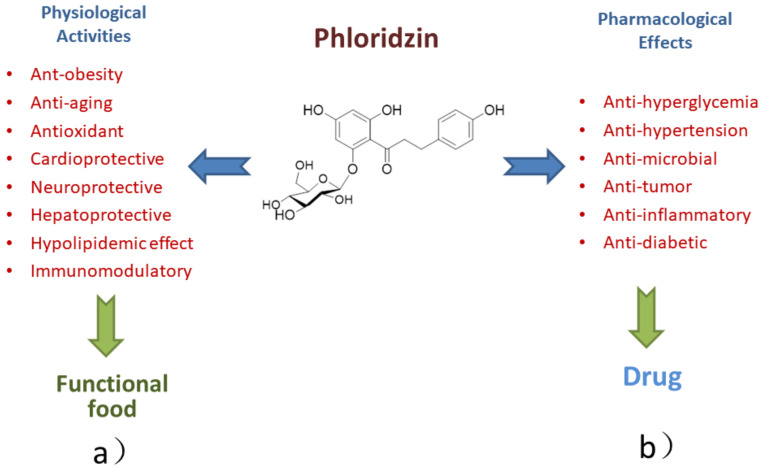
The potential applications and further use of phloridzin: (**a**) as an active compound in functional food and (**b**) as an active pharmaceutical ingredient in the pharmaceutical industry after optimization.

These studies showed that phloridzin had multiple pharmacological activities, such as antidiabetic, anti-inflammatory, antihyperglycemic, anticancer and antibacterial activities. Besides, the physiological effects of phloridzin are cardioprotective, neuroprotective, hepatoprotective, immunomodulatory, antiobesity, antioxidant activities and so on (Table 4, Figure 2).

## 5. Future Perspectives

Phloridzin, a kind of natural organic compound, exists in numerous fruits and plants, and has been investigated for a long time because of its extensive bioactivities. Phloridzin was first used as a treatment for fever, infectious diseases and malaria about 180 years ago. Later, it played a key role in renal glucose reabsorption and hyperglycemia of diabetes. Phloridzin exerted an effective inhibitory activity on both SGLT1 and SGLT2, which made it widely used in medicine and physiological research. Although phloridzin holds potential for the treatment of diabetes, it has some limitations, including poor intestinal absorption, low bioavailability, rapid degradation by β-glucosidase and lactose–phloridzin–hydrolase and several adverse effects in clinical trials [108]. Although phloridzin itself was not developed as an antidiabetic drug, several phloridzin analogs (e.g., dapagliflozin and empagliflozin) show much more pharmacological viability, and these have been studied as therapeutic agents for diabetes [8]. In future, more investigations are needed on the various pharmacological mechanisms of phloridzin analogs and the biological activities of phloridzin metabolites.

Other studies suggest that phloridzin holds potential for food additives, cosmetics, beverages and food preservatives. Phloridzin is also a prospective biomarker for the consumption of phloridzin-containing foods. Phloridzin contributes to the characteristic taste of ciders, when its concentration is within the range of 3–16 g/L, while natural dimerized phloridzin plays important roles in the color formation of apple juices and ciders [109]. Therefore, there is a necessity for more studies to elucidate the toxicokinetics and toxicodynamics related to phloridzin and its metabolites in food applications. Moreover, how to apply drug packaging to phloridzin to improve its bioavailability should be paid more attention.

## Figures and Tables

**Table 1:** Phloridzin-containing plant species.

Plant species	Tissue	Extraction Solvent	References
*Acanthopanax senticosus*	Root	Methanol	[17]
*Acca sellowiana*	Flesh	Methanol	[18]
*Aspalathus linearis*	Leaf	Methanol and Water (1% Formic acid)	[19]
*Aspalathus pendula*	Leaf	Methanol and Water (1% Formic acid)	[19]
*Berberis jaeschkeana*	Flesh	Methanol	[20]
*Camellia japonica*	Leaf	Water	[21]
*Docynia dcne*	Leaf	Methanol and Acetonitrile	[22]
*Docynia indica*	Leaf	Ethyl acetate, n-hexane, n-butanol and Methanol	[23]
*Eleutherococcus senticosus*	Root	Methanol	[24]
*Fragaria x ananassa*	Flesh	Acetone	[25]
*Glycine max*	Seed	Ethanol	[26]
*Hemerocallis x hybrida*	Flower	Hexane, Ethyl acetate and Methanol	[27]
*Lactuca sativa*	Flesh	Water	[28]
*Lithocarpus polystachyus*	Leaf	Ethanol, Methanol	[29,30,31,32]
*Lippia graveolens*	Leaf	Methanol	[33]
*Nelumbo nucifera*	Seed	Ethanol	[34]
*Pisum sativum*	Seed	Acetone	[35]
*Polygonum cuspidatum*	Flower	Methanol	[36]
*Prunus persica*	Flesh	Methanol and Water with Formic acid	[37]
*Psidium guajava*	Peel and Flesh	Methanol and Water with Formic acid	[38]
*Punica granatum*	Flesh	-	[39]
*Pyrus pashia*	Flesh	Ethanol	[40]
*Rosa canina*	Flesh	Methanol and Water with Formic acid	[41]
*Rubus occidentalis*	Flesh	Methanol	[42]
*Solanum lycopersicum*	Flesh	Methanol	[43]
*Sechium edule*	Flesh	Methanol	[44]
*Vaccinium vitis-idaea*	Flesh	Methanol, Ethanol, Formic acid, Acetone, Ethyl acetate, Water,	[45]
*Vaccinium macrocarpon*	Flesh	Methanol	[46]

**Table 2:** Antioxidant effects of phloridzin.

Plant Species	Tissue	Experimental Assays	References
*Eleutherococcus senticosus*	Root	2,2-diphenyl-1-picrylhydrazyl (DPPH)	[24]
*Malus domestica*	Peel	Thiobarbituric acid reactive substance (TBARS) assay	[72]
*Malus domestica*	Leaf	2,2-azino-bis(3-ethylbenzothiazoline-6-sulphonic acid) (ABTS), DPPH, Ferric-reducing antioxidant power (FRAP)	[70]
*Malus domestica*	Flesh	ABTS, DPPH	[69]
*Malus domestica*	Flesh (thinned young)	TBARS	[75]
*Malus toringoides*	Leaf	ABTS, DPPH, FRAP	[74]
*Nelumbo nucifera*	Seed	ABTS, DPPH, FRAP	[34]
		TBARS	[71]
		Paraquat challenge assay, H_2_O_2_ challenge assay, enzyme activity assay	[73]

**Table 3:** Anti-inflammatory effects of phloridzin.

Experimental Model	Inflammatory Index Assays	References
Diet-Induced Obese Mice	Tumor Necrosis Factor-α (TNF-α), Monocyte Chemoattractant Protein-1 (MCP-1), Interferon-γ (IFN-γ), Interleukin-6 (IL-6), Leptin, Adipsin	[76]
LPS-Stimulated RAW 264.7 Cells	Nitric Oxide (NO), Inducible Nitric Oxide Synthase (iNOS), TNF-α, IL-10	[77]
IL-1β-Stimulated Myofibroblasts	Prostaglandin E2 (PGE_2_), Intercellular Cell Adhesion Molecule-1 (ICAM-1), IL-8, IL-6, MCP-1	[78]
LPS-Stimulated RAW 264.7 Cells	Cyclooxygenase-2 (Cox-2), NO, PGE2, IL-6, TNF-α, iNOS	[79]
UVB-Induced Mouse Skin Damage, UVB-Induced HaCaT Cells	Reactive Oxygen Species (ROS), IL-1β, IL-6, IL-8, Cox-2	[80]
Diet-Induced Obese Mice	MCP-1, TNF-α, IL-1, IL-6, IL-1β	[81]

**Table 4:** Therapeutic properties of phloridzin.

Type	Therapeutic Properties	Methods	Main Findings	References
Pharmacological effects	Antihypertension	Phloridzin (0.4 g/kg body weight/day) was given for 4 weeks in propylene glycol solution (20%) by subcutaneous administration.	Phloridzin treatment prevented the development of hypertension, decreased SGLT2 activity in diabetic rats.	[62]
	Antidiabetic	Phloridzin (purity > 98%) was given daily in sterile saline solution (20 mg/kg body weight) by intragastric administration.	Phloridzin normalized hyperglycemia of type 2 diabetic (db/db) mice by serum lipopolysaccharide decrease and gut microbiota change.	[66]
	Antihyperglycemic	Phloridzin (100 mg) was isolated from the leaves of *Malus hupehensis* by preparative HPLC with elution 30% acetonitrile (ACN) and 0.1% acetic acid (HAc).	Phloridzin showed significant concentration-dependent inhibitory effects on α-glucosidase.	[68]
	Anti-inflammatory	Phloridzin (16.4–84.11 and 6.6–45.1 µg/g formula weight (FW) in peel and flesh, respectively) was identified by high-performance liquid chromatography-diode array detector-tandem mass spectrometry (HPLC-DAD-MS/MS) analysis in the peel and flesh of different apple cultivars.	Phloridzin improved the quality of anti-inflammatory response at the intestinal level and, accordingly, ameliorated cytokine-driven inflammation.	[78]
	Antitumor	Phloridzin was isolated from the leaves of *Malus* crabapples by preparative HPLC.	3-(4,5-dimethylthiazol-2-yl)-2,5-diphenyl tetrazolium bromide (MTT) cancer cell growth inhibition assay demonstrated that phloridzin from crabapple leaves had an antitumor effect.	[87]
	Antimicrobial	Phloridzin (above 500 mg per g of ethyl acetate extract from the leaves of *Malus domestica*) was identified by HPLC.	Antimicrobial activity was observed for ethyl acetate extract against strains of *Staphylococcus aureus* ATCC 25923, *Enterococcus faecalis* ATCC 29212 and *Candida glabrata* ATCC 90030.	[88]
Physiological activities	Neuroprotective	Phloridzin (3.0–300.0 µg/kg) was dissolved in saline before being used and was given intraperitoneally.	Phloridzin influenced memory storage, but not memory retrieval.	[57]
	Antioxidant	Phloridzin (534 ± 8.31 μg/g) in a crude extract of E Se tea was identified and quantified using a ultra-high-performance liquid chromatography coupled with tandem mass spectrometry (UHPLC-ESI-HR-MS/MS).	Phloridzin significantly prevented oxidative stress damage and reduced the apoptosis of H_2_O_2_-induced HepG2 cells.	[74]
	Antiobesity	Male C57BL/6J mice were fed a high-fat diet with phloridzin (0.02%, *w*/*w*) for 16 weeks.	Supplementation of phloridzin ameliorated not only insulin resistance, but also obesity in high-fat diet-induced obese mice.	[76]
	Hypolipidemic	Phloridzin (purity >98%, 20 mg/kg) was administered in normal saline solution by intragastric administration for 10 weeks.	Phloridzin decreased body weight gain and the levels of glucose, blood total cholesterol (TC) and blood triglycerides (TG) in blood and, accordingly, ameliorated hepatic damage of type 2 diabetic mice.	[82]
	Hepatoprotective	Phloridzin (40 mg/kg/day) and phloridzin (80 mg/kg/day) was given orally for 10 consecutive days. At the end of day 3 of the experiment, the rats were administered methotrexate.	Phloridzin protected against hepatic injury in rats mainly through mitigation of oxidative stress, inflammation and apoptosis in hepatic tissues.	[83]
	Cardioprotective	Phloridzin (purity >98%) was dissolved in normal saline solution and administered intragastrically from week 8 to week 18 without hypoglycemic therapy.	Phloridzin prevented the development of diabetic cardiomyopathy by regulating the expression of key proteins in these processes.	[91]
	Antiaging	Phloridzin (4.0 g) was isolated from branches of dwarf apple JM7 (4.0 kg), and the chemical structure was determined by comparing ^1^H and ^13^C NMR spectra.	Phloridzin (10 mM, 30 mM) had an antiaging effect on yeast by increasing the activity of superoxide dismutase (SOD) and SIRT1.	[99]
	Immunomodulatory	Extracts from the fruits of *Malus baccata* contained phloridzin (relative content 18.24%).	The extracts had radioprotective and immunomodulatory activities.	[103]
